# Artificial Intelligence in Molecular Optimization: Current Paradigms and Future Frontiers

**DOI:** 10.3390/ijms26104878

**Published:** 2025-05-19

**Authors:** Xin Xia, Yajie Zhang, Xiangxiang Zeng, Xingyi Zhang, Chunhou Zheng, Yansen Su

**Affiliations:** 1 The Key Laboratory of Intelligent Computing and Signal Processing of Ministry of Education, School of Artificial Intelligence, Anhui University, Hefei 230601, China; xiaxin98@stu.ahu.edu.cn; 2 The Key Laboratory of Intelligent Computing and Signal Processing of Ministry of Education, School of Computer Science and Technology, Anhui University, Hefei 230601, China; yjzhang17719490727@163.com (Y.Z.); xyzhanghust@gmail.com (X.Z.); zhengch99@126.com (C.Z.); 3 College of Computer Science and Electronic Engineering, Hunan University, Lushan Road, Changsha 410012, China; xzeng@hnu.edu.cn

**Keywords:** molecular optimization, artificial intelligence, iterative search, end-to-end generation

## Abstract

Molecular optimization plays a pivotal role in many domains since it holds promise for improving the properties of lead molecules. The advent of artificial intelligence (AI)-driven molecular optimization has revolutionized lead optimization workflows, which have significantly accelerated the development of drug candidates. However, AI models are also confronted with new challenges in practical molecular optimization, such as high-dimensional chemical space and data sparsity issues. This paper initially highlights the inherent benefits of molecular optimization in terms of optimizing the properties and maintaining the structural similarity of lead molecules, thereby highlighting its critical role in drug discovery. The next section systematically categorizes and analyzes existing AI-aided molecular optimization methods, comprising iterative search in discrete chemical space, end-to-end generation in continuous latent space, and iterative search in continuous latent space methods. Finally, we discuss the key challenges in AI-aided molecular optimization methods, including molecular representations, dataset selection, the properties to be optimized, and optimization algorithms, while proposing potential solutions and future research directions. In summary, this review provides a comprehensive analysis of existing representative AI-aided molecular optimization methods, thereby offering guidance for future research directions.

## 1. Introduction

In various engineering fields such as materials engineering, the chemical industry, and drug development, molecular optimization plays a pivotal role in enhancing molecular properties by modifying the structures of lead molecules [1,2]. The drug discovery pipeline comprises several critical stages, including target identification, lead compound screening, lead compound optimization, and preclinical and clinical validation, which is a time-consuming and expensive process with a high failure rate [3]. The inherent complexity of human pathophysiology, coupled with the vastness of chemical space, necessitates rigorous decision-making at each stage of the discovery process. Thus, it is imperative to employ specific techniques to accelerate the drug discovery process and enhance the success rate [4,5]. Computer-aided drug design has significantly advanced various key aspects of drug discovery through its applications in disease target prediction, virtual screening, molecular optimization, etc. [6,7,8]. Among these applications, molecular optimization is one of the crucial steps in obtaining optimal drug candidates, with the aim of improving the properties of lead molecules, such as their biological activity properties and physicochemical properties, while preserving their structural features [9,10]. The strategic optimization of the unfavorable properties of lead molecules significantly increases their likelihood of success in subsequent preclinical and clinical evaluations [11,12]. Therefore, the development of efficient molecular optimization methods offers substantial potential for streamlining the drug discovery and development process.

In recent years, artificial intelligence (AI)-aided molecular optimization methods have been extensively developed, facilitating a more comprehensive exploration of the huge chemical space and holding promise for enhancing the drug discovery and development process [13,14,15]. For example, while conventional drug development takes around 12 years and costs USD 2.6 billion on average [16], Zhavoronkov et al. proposed a deep learning model to rapidly identify DDR1 kinase inhibitors in just 21 days, substantially reducing both time and cost [17]. In general, AI-based molecular optimization methods follow two processes: the selection or construction of appropriate chemical spaces, followed by the exploration of the space to identify target molecules. Within this framework, many combinatorial optimization techniques operating directly on discrete molecular representations, such as molecular sequences and graphs, have been proposed to optimize molecules. Furthermore, the integration of deep learning has introduced novel capabilities, enabling the construction of continuous latent spaces for chemical molecules. This advanced representation facilitates molecular optimization through continuous vector space manipulation, offering an alternative to traditional discrete optimization approaches.

However, AI-aided molecular optimization methods face both significant challenges and emerging opportunities in practical drug discovery applications. The primary limitation stems from the inherent constraints of conventional molecular representations, which bring different challenges for effective AI-driven optimization [18]. Moreover, the efficacy of these AI-driven methods is fundamentally contingent upon the availability and quality of relevant molecular datasets [19]. Well-curated datasets are essential for training optimization models and ensuring their applicability. Furthermore, practical molecular optimization must navigate the complex task of simultaneously enhancing multiple properties while maintaining critical structural constraints [20]. This multi-objective optimization problem necessitates the development of more efficient computational frameworks. In addition, the rapid advancement of AI technologies offers promising avenues for addressing these challenges. Novel machine learning architectures and optimization algorithms hold significant potential for developing more efficient and reliable molecular optimization methods [21].

In this review, we provide a comprehensive overview of AI-aided molecular optimization. We first emphasize the advantages of molecular optimization to highlight its significance in drug discovery. Then, we summarize recent AI-aided molecular optimization methods, categorizing them into two distinct paradigms: combinatorial optimization methods operating in discrete chemical space and deep learning models operating in continuous latent space. Furthermore, we compile the experimental results of various methods on the same experimental task to visually demonstrate the optimization performance of different approaches. Finally, we discuss the challenges associated with AI-aided molecular optimization in practical drug discovery and offer corresponding recommendations. Our aims are to provide insights into AI-aided molecular optimization and offer guidance for future directions in computational drug discovery.

## 2. Definition of Molecular Optimization

In the drug discovery process, molecular optimization represents a critical stage subsequent to the lead molecule screening stage, which focuses on the structural refinement of promising lead molecules to enhance their properties. Therefore, molecular optimization methods can optimize specific properties of a given molecule, leading to molecules with enhanced properties. For example, Jin et al. [22] established a benchmark optimization task that requires improving molecules with quantitative estimation of drug-likeness (QED) values ranging from 0.7 to 0.8 to achieve QED scores exceeding 0.9 while maintaining a structural similarity value larger than 0.4. It is worth noting that, compared to de novo molecular generation, molecular optimization beginning with the lead molecule can shorten the search process for finding target molecules. The definition of molecular optimization is formulated as follows:

**Definition** **1.** *Given a lead molecule x, its associated properties are $p_{1}\left(x\right),\ldots ,p_{m}\left(x\right)$, and the goal of molecular optimization is to generate a molecule y with properties $p_{1}\left(y\right),\ldots ,p_{m}\left(y\right)$, satisfying*

(1) $$\left\{\begin{array}{c} p_{i}\left(y\right)≻p_{i}\left(x\right),i=1,2,\ldots ,m,\\ sim(x,y)>\delta , \end{array}\right.$$

*where $p_{i}\left(y\right)≻p_{i}\left(x\right)$ indicates that $p_{i}\left(y\right)$ is better than $p_{i}\left(x\right)$. $p_{i}$ represents a molecular property, which can encompass various physicochemical and pharmacological properties, such as QED, bioactivity, and synthetic accessibility. sim(x,y) is the similarity between x and y, and δ is the threshold of similarity. A frequently used molecular similarity metric is the Tanimoto similarity [23] of Morgan fingerprints [24], which is shown in Equation (2):*

(2) $$sim\left(x,y\right)=\frac{fp(x)\cdot fp(y)}{{\left|fp(x)\right|}^{2}+{\left|fp(y)\right|}^{2}-fp\left(x\right)\cdot fp\left(y\right)},$$

*where fp represents the Morgan fingerprints of the molecule. A fundamental consideration in molecular optimization is the necessity of maintaining structural similarity between the optimized molecule and its lead compound. This similarity constraint serves a dual purpose. First, it effectively delineates the chemical space to be explored around the lead molecule, thereby enhancing search efficiency [25]. Second, it preserves crucial structural features that are essential for maintaining desirable physicochemical and biological properties [26]. The significance of structural similarity is reflected in its incorporation into numerous benchmark molecular optimization tasks. For example, one widely adopted benchmark task involves optimizing the penalized logP of molecules while maintaining a Tanimoto similarity larger than 0.4 [22]. Another extensively studied benchmark task aims to improve biological activity against the dopamine type 2 receptor (DRD2) while preserving a structural similarity value greater than 0.4 [22].*

## 3. Current Approaches and Barriers

**Figure 1:**
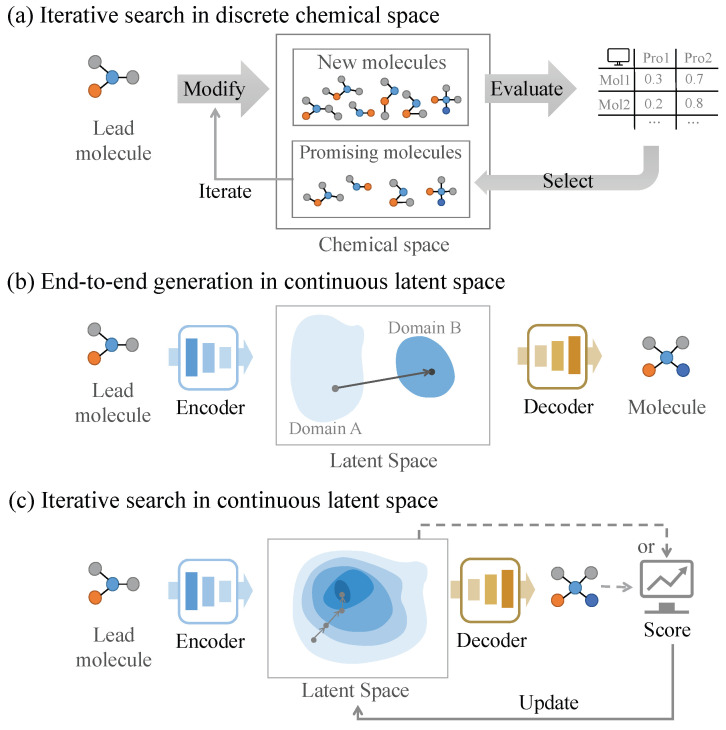
The workflows of artificial intelligence models for molecular optimization.

AI-aided molecular optimization methods typically involve two fundamental steps: (1) the construction of an implicit chemical space and (2) the implementation of an optimization approach to find the desired molecules within the implicit chemical space. Existing AI-aided molecular optimization methods can be broadly classified based on their operational spaces: discrete chemical spaces and continuous latent spaces. For discrete chemical space approaches, molecules are represented through discrete structural representations, such as molecular sequences or graph-based structures, enabling direct structural modifications. Conversely, continuous latent space methods employ encoder–decoder frameworks to transform molecules into continuous vector representations, facilitating optimization in a differentiable space. To systematically organize these methods, this section categorizes these methods based on the constructed chemical spaces and the employed optimization algorithms. For an enhanced comparative analysis, Figure 1 shows the workflows of various AI-based molecular optimization methods, and Table 1 provides a comprehensive summary of representative AI-based molecular optimization methods, along with their molecular representations, data types, and optimization objectives.

### 3.1. Molecular Optimization in Discrete Chemical Spaces

Molecular optimization methods operating in discrete chemical spaces employ direct structural modifications based on discrete representations, such as SMILES [27], SELFIES [28], and molecular graphs [29] (where nodes represent atoms, and edges represent chemical bonds). These methods typically explore the discrete chemical space through the following process: First, they generate a set of novel molecular structures through structural modifications, and then they select promising molecules for subsequent iterative optimization, as illustrated in Figure 1a. These methods can be primarily classified into genetic algorithm (GA)-based methods and reinforcement learning (RL)-based methods.

#### 3.1.1. GA-Based Molecular Optimization Methods

Genetic algorithms (GAs) are heuristic optimization approaches that show competitive optimization performance to explore chemical spaces globally and locally. GA-based optimization methods begin with an initial population and generate new molecules through crossover and mutation operations. Then, molecules with high fitness are selected in the new population to guide the evolution process [30]. Some GA-based molecular optimization methods only mutate molecules while maintaining structural similarity. For example, STONED [31] generates offspring molecules by applying a random mutation on the SELFIES strings of molecules, which finds molecules with better properties. However, the absence of a crossover operator limits the global exploration of the vast chemical space. MolFinder [32] integrates crossover and mutation in the SMILES-based chemical space, which enables both global search and local search. All of the aforementioned methods aggregate multiple properties into a single fitness function to guide the evolution, which requires predefined weights for multiple properties. In comparison, GB-GA-P [33] employs two Pareto-based genetic algorithms on molecular graphs, thereby enabling multi-objective molecular optimization to identify a set of Pareto-optimal molecules with enhanced properties.

In short, GAs have gained widespread adoption in molecular optimization due to their inherent flexibility, robustness, and ability to explore chemical space without requiring extensive training datasets. However, the efficacy of GA-based molecular optimization methods depends on the population size and the number of evolutionary generations, since repeated evaluations of molecular properties can be costly.

**Table 1:** Representative molecular optimization methods and their categories, molecular representations, data types, and optimization objectives.

Category	Model	Molecular Representation	Data Type	Optimization Objective	Citation
Iterative search in discrete space	STONED	SELFIES	Unpaired	Multi-property	[31]
	MolFinder	SMILES	Unpaired	Multi-property	[32]
	GB-GA-P	Graph	Unpaired	Multi-property	[33]
	GCPN	Graph	Unpaired	Single-property	[34]
	MolDQN	Graph	Unpaired	Multi-property	[35]
End-to-end generation in continuous space	CMG	SMILES	Paired	Multi-property	[36]
	T&S polish	Graph	Paired	Multi-property	[25]
	Mol-CycleGAN	Graph	Unpaired	Single-property	[37]
	UGMMT	SMILES	Unpaired	Single property	[38]
	IPCA	SMILES	Unpaired	Multi-property	[39]
	GPMO	SMILES	Paired	Multi-property	[40]
	VJTNN	Graph	Paired	Single-property	[22]
	SCVAE	Graph	Paired	Single-property	[41]
	Modef	Graph	Paired	Multi-property	[42]
	CFOM	SMILES	Unpaired	Single-property	[43]
	TamGen	SMILES	Unpaired	Single-property	[44]
Iterative search in continuous space	QMO	SMILES	Unpaired	Multi-property	[45]
	DST	Graph	Unpaired	Multi-property	[46]
	LIMO	SELFIES	Unpaired	Multi-property	[47]
	InversionGNN	Graph	Unpaired	Multi-property	[48]
	MOMO	SMILES	Unpaired	Multi-property	[49]
	DecompOpt	3D	Unpaired	Multi-property	[50]
	GCDM	3D	Unpaired	Multi-property	[51]
	Retmol	SMILES	Unpaired	Multi-property	[52]
	MO-LSO	Graph	Unpaired	Multi-property	[53]
	Prompt-MolOpt	SMILES	Paired	Multi-property	[54]
	Drugassist	SMILES	Unpaired	Multi-property	[55]

#### 3.1.2. RL-Based Molecular Optimization Methods

Reinforcement learning (RL) [56] is a machine learning paradigm used to address decision-making problems, and it has shown potential in optimizing molecular properties by designing states, actions, and rewards. Most RL-based molecular optimization methods operate on molecular graphs. For example, GCPN [34] formalizes molecular optimization as a Markov decision process, which modifies molecules by adding atoms or fragments and connecting them with bonds. Additionally, GCPN uses a policy network to predict actions, which integrates molecular properties and an adversarial loss as rewards to update the policy gradients. In comparison, MolDQN [35] directly applies actions to the molecular graph and ensures molecular validity by chemically valid actions. MolDQN trains a Deep Q network to estimate the rewards, which enables it to discover molecules with enhanced properties.

Overall, RL-based molecular optimization methods facilitate active exploration of the chemical space beyond the training data. These approaches typically define the molecular modification process as a Markov process that performs sequential modifications to refine the molecular structure. However, the iterative modification can be inefficient due to the large number of available substructures in chemical space.

#### 3.1.3. Analysis of Molecular Optimization Methods in Discrete Space

GA-based molecular optimization methods exhibit strong flexibility and broad applicability. By leveraging population-based parallel search mechanisms, GAs can explore a wider chemical space while reducing the risk of converging to local optima. These methods only require molecular property evaluators to compute the fitness function, significantly reducing the dependence on labeled data. Consequently, they demonstrate superior task scalability and can be flexibly applied to various quantifiable molecular optimization scenarios. In contrast, RL-based molecular optimization methods maximize the global reward through interactions between actions and reward environments. However, RL typically requires a large number of iterations to converge, meaning that obtaining high-quality optimized molecules often demands substantial computational resources. It is worth noting that molecular optimization based on discrete representations has certain limitations. It relies on expert-designed modification rules. The generated molecular structures could be chemically invalid. The exploration efficiency of chemical space is relatively limited.

### 3.2. Molecular Optimization in Continuous Latent Spaces

The rapid advancement of deep learning (DL) techniques has opened up new opportunities for molecular optimization. DL-based molecular optimization methods leverage the powerful nonlinear representation capabilities of deep neural networks to extract complex chemical knowledge from extensive molecular datasets, thereby facilitating the construction of continuous latent spaces. These methods typically employ an encoder–decoder framework, where an encoder transforms discrete molecules into continuous latent space, which enables them to efficiently modify the continuous vector of the lead molecule to obtain new vectors, and a decoder maps these new vectors back to discrete chemical space to obtain novel molecular structures with enhanced properties. In this subsection, we categorize molecular optimization methods in continuous latent spaces into end-to-end generation methods and iterative search methods.

#### 3.2.1. End-to-End Generation Methods

End-to-end generation molecular optimization methods typically employ a deep learning architecture comprising an encoder–decoder framework (Figure 1b). These methods directly generate optimized molecular structures as output through the input of a lead molecule, and they can be further classified into translation-based methods and conditional generation-based methods. Translation-based methods learn the translation rules from matched molecular pairs or sets, which enables the model to map input lead molecules to their optimized structures. Conditional generation-based methods integrate additional condition features (e.g., target properties or structural constraints) with the lead molecule to guide the generation of novel molecular structures with desired properties.

**Translation-based methods.** Inspired by the conceptual analogy between molecular optimization and translation tasks in natural language processing, many translation-based molecular optimization methods have been proposed to facilitate the transformation of lead molecules into target molecules [57]. For example, CMG [36] treats molecular optimization as a sequence-to-sequence translation problem, and it employs a Transformer framework with two constraint networks to generate structurally similar molecules based on SMILES. This approach relies heavily on molecular sequence representation and the Transformer architecture. In comparison, Graph Polish [25] adopts molecular graph representation, which translates lead molecules to optimized molecules through two modules, i.e., a pre-labeling module and a translation module. To be specific, the pre-labeling module identifies the optimization centers and label branches in the molecules, while the translation module trains a deep neural network from the labeled molecules to translate the target molecules. The graph-based approach emphasizes the structural integrity and topological features of molecules.

While most translation-based molecular optimization methods rely on paired molecules for supervised learning, several unsupervised translation-based methods have been developed to address the challenge of limited paired data. For example, Mol-CycleGAN [37] leverages the CycleGAN framework in the latent space of the JT-VAE codec [58], which divides the training data into low- and high-property domains to facilitate adversarial learning across these two domains. Similarly, UGMMT [38] employs CycleGAN to learn the translation rules based on molecular SMILES representations. Although both Mol-CycleGAN and UGMMT can translate lead molecules to target molecules with improved properties, their optimization capabilities are limited to a single molecular property. In comparison, IPCA [39] extends UGMMT by introducing an integrated poly-cycle architecture that concurrently optimizes multiple properties. This approach translates molecules through a shared latent embedding space and a central decoder, thereby allowing for the optimization of two properties. Additionally, translation-based methods also face challenges such as exposure bias, where the generation of molecules depends on the previously predicted outputs [59]. To mitigate this problem, GPMO [40] integrates contrastive learning into the Transformer framework to translate desired molecules while reducing exposure bias.

In summary, translation-based molecular optimization methods learn transition rules from matched molecular pairs or sets, which enables end-to-end optimization by directly generating optimized molecules from input lead molecules. However, these methods face notable limitations, particularly the scarcity of molecular data that simultaneously satisfy the multiple property conditions required for effective model training. Furthermore, although transformation rules can be inferred from matched molecular sets categorized by low and high property values, the lack of explicit structural guidance may impede the optimization process.

**Conditional generation-based methods.** Several molecular optimization methods generate molecules with enhanced properties by integrating the features of lead molecules with specific conditions, such as the structures or properties of the target molecules. For example, VJTNN [22] employs a graph message passing network to encode both the molecular graphs and junction trees of paired molecules, i.e., the lead molecule and its corresponding target molecule. The features of the target molecule are extracted as conditions, which are subsequently fused with the latent vector of the lead molecule to generate new molecules. Later on, SCVAE [41] leverages the graph alignment for paired molecules, which incorporates structural similarity as a condition during the decoding process to produce target molecules. However, both VJTNN and SCVAE require the encoding and decoding of entire molecular graphs, which introduces significant learning challenges due to computational complexity and data requirements.

In contrast, Modef [42] simplifies this process by encoding only the differences between paired molecular graphs as conditional inputs. This approach not only reduces the number of parameters but also minimizes the amount of training data required, thereby enhancing computational efficiency and scalability. CFOM [43] decomposes the lead molecule into a molecular core and molecular chains. Utilizing a core encoder and a chains generator, CFOM generates novel chains, which are subsequently attached to the core to produce new molecules with enhanced properties. Furthermore, in recent years, several studies have used the structure of target proteins to generate target-aware molecules. For example, the TamGen framework [44] processes the geometric data of amino acids to generate protein representations while simultaneously incorporating molecular SMILES to derive molecular embeddings. The protein representation is subsequently utilized as a conditional to output optimized molecules by a compound decoder.

Conditional generation-based molecular optimization methods generate optimized molecules by incorporating specific conditions on properties or structures. These methods generate high-quality molecules by leveraging the conditions to guide the optimization process. However, a notable limitation of these approaches is the prerequisite of obtaining the target conditions prior to model training.

#### 3.2.2. Iterative Search Methods

Iterative search-based molecular optimization methods in continuous latent space typically explore the space through step-by-step optimization to identify superior molecular continuous vectors (Figure 1c). When iterative search-based methods generate a set of molecules, these molecules are evaluated and selected to update the molecular continuous vector or to retrain the generator model for iterative optimization. There are several representation iterative search-based methods, which are introduced below.

For example, QMO [45] decouples the molecular representation learning and the guided search processes by using a pre-trained encoder–decoder framework. This framework evaluates molecular properties in discrete space and approximates gradients in continuous space by a model-independent zero-gradient descent method. However, the accuracy of the approximated gradients can significantly impact the search process. There are some methods that compute gradients based on the property values predicted in continuous latent spaces. For example, DST [46] trains graph neural networks on molecular differentiable scaffold tree representation to predict properties, which updates the scaffold tree of molecules by propagating local derivatives. In addition, LIMO [47] integrates a SELFIES-based VAE with a property prediction network, which facilitates rapid gradient-based optimization. InversionGNN [48] is a sample-efficient, dual-path graph neural network (GNN)-based framework designed for multi-objective molecular optimization. In its direct prediction path, InversionGNN leverages a GNN to extract knowledge from differentiable molecular scaffolding trees, enabling accurate property prediction. Subsequently, it employs gradient-based Pareto optimization to approximate molecules along the Pareto front.

There are several iterative search-based methods that explore the continuous latent space without updating the gradient. For example, MOMO [49], combines a pre-trained encoder–decoder with a Pareto-based evolutionary algorithm to collaboratively evolve molecules between implicit space and discrete space. DecompOpt [50] uses diffusion models to capture molecular grammar in a data-driven manner, and it integrates iterative optimization to generate molecules with desired properties. Similarly, Morhead et al. [51] developed a geometry-complete diffusion model (GCDM), which learns the essential geometric properties of 3D molecules, enabling the generation of valid 3D molecular structures. The GCDM achieves property-guided 3D molecular optimization by iteratively accepting the generated molecules as intermediate states.

There are also existing methods that iteratively update the database for search-based optimization. For example, Retmol [52] samples high-quality molecules from a predefined retrieval dataset, which are combined with the lead molecule to obtain optimized molecules. The generated molecules are dynamically added to the retrieval database for iterative optimization. MO-LSO [53] employs an iterative weighted retraining strategy, which progressively refines the generative model to generate desired molecules. Specifically, MO-LSO performs Pareto ranking on the training molecules and assigns weights for these molecules based on their ranks. Then, it trains the generative model based on the weighted dataset to produce enhanced molecules. The newly generated molecules are ranked to update the training set, which is further used to refine the generative model.

In addition, recently, the advent of large language models (LLMs) has spurred their application in molecular optimization. For example, Prompt-MolOpt [54] integrates large language models (LLMs) with Transformer architectures to enhance molecular optimization capabilities. It employs an iterative fragment-based optimization strategy; i.e., at each step, a single molecular substructure is modified, which has demonstrated potential in multi-property molecular optimization. DrugAssist [55] is an interactive molecular optimization framework that iteratively refines molecular structures through human–AI dialogue. After an optimized molecule is generated by DrugAssist, its properties are evaluated. If the molecule meets the predefined property requirements, the process terminates. If not, DrugAssist retrieves molecules from the database that are the most structurally similar to the lead molecule and satisfy the property constraints, guiding further optimization until the desired molecular properties are achieved.

In summary, iterative search-based molecular optimization methods mitigate the reliance of deep learning models on extensive training data by incrementally identifying molecules with enhanced properties through a step-by-step optimization process. However, this iterative nature inherently renders these methods more computationally intensive and time-consuming.

#### 3.2.3. Analysis of Molecular Optimization Methods in Continuous Space

Compared to molecular optimization in discrete chemical space, the construction of continuous chemical spaces enables a more efficient and smooth exploration of high-quality molecules [60]. Among continuous-space approaches, end-to-end generation methods offer faster optimization speeds. Once the models are trained on relevant datasets, they can perform batch molecular optimization in a single forward step. However, the optimization capability of end-to-end methods heavily depends on the quality of the training data and the training process [61]. When the training data are limited, the optimized molecules tend to exhibit relatively lower property values. Iterative search in continuous space has emerged as a popular new paradigm in molecular optimization in recent years. These methods require fewer labeled target molecules for training and can search for better molecules through iterative property evaluation. However, they typically involve multiple rounds of iterative optimization for a single lead molecule, resulting in longer optimization times.

### 3.3. Optimization Performance Comparison

To systematically evaluate the optimization performance of different AI-based molecular optimization methods, this study selected three representative benchmark tasks and integrated results from the literature with partially reproduced experimental data. The experimental designs for the three optimization tasks are as follows:

Task 1: PlogP optimization task

The objective of Task 1 is to maximize the penalized logP (PlogP) property value of molecules while maintaining a Tanimoto similarity of at least 0.4 with the lead molecules. The experiment uses the benchmark dataset constructed by Jin et al. [22], which consists of 800 molecules with low PlogP values selected from the ZINC database. The evaluation metric for this task is the average PlogP improvement of the optimized molecules.

Task 2: QED optimization task

The goal of Task 2 is to improve the QED of molecules while preserving a similarity value of at least 0.4 with the lead molecules. The test set proposed by Jin et al. [22] contains 800 molecules with QED values ranging from 0.7 to 0.8. The evaluation metric for this task is the optimization success rate, defined as the proportion of lead molecules whose QED is improved to above 0.9 while maintaining a similarity value larger than 0.4 among all tested lead molecules.

Task 3: Multi-property optimization task

Task 3 involves optimizing four properties: QED, synthetic accessibility (SA), the estimated inhibition score against the glycogen synthase kinase-3β target (GSK3β inhibition), and the estimated inhibition score against the c-Jun N-terminal kinase-3 target (JNK3 inhibition). The evaluation metric is the average property score (APS) of the top 100 generated molecules.

**Figure 2:**
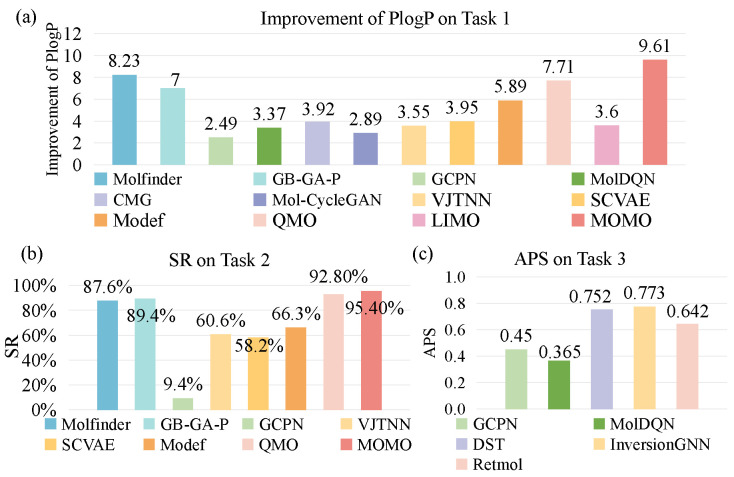
The performance of some existing methods on three optimization tasks. (**a**) The average PlogP improvement on Task 1. (**b**) The success rate (SR) on Task 2. (**c**) The average property score (APS) on Task 3.

Figure 2 presents a performance comparison of different molecular optimization methods across three benchmark tasks. To ensure experimental fairness, we adopted uniform evaluation criteria and clearly state the source of each result. In Figure 2a, the average property improvement values of twelve methods in the PlogP optimization task are displayed. In the figure, the results for MolFinder, GB-GA-P, and MOMO were obtained by our reproduction of the experiments under the same oracle call settings, while the other results were extracted from the original publications. The results show that the iterative search methods achieved superior PlogP improvement while maintaining molecular similarity. Figure 2b compares eight methods in the QED optimization task. The MolFinder and GB-GA-P results came from our reproduction experiments with matched oracle calls, and the others were sourced from the original papers. The iterative search methods again showed better performance in QED optimization. Figure 2c presents the results of five methods on Task 3, all of which are cited from the original publications. Among these methods, InverseGNN exhibited the best comprehensive optimization across all four properties. Notably, due to architectural differences between the models, the training datasets varied across the methods (detailed in the original references). All reproduction experiments strictly followed the hyperparameter settings recommended in the original papers.

## 4. Crucial Considerations and Future Opportunities

### 4.1. Reasonable Molecular Representation

Molecular optimization relies on several widely used molecular representations, the quality of which significantly influences the performance of molecular optimization methods. In this section, we outline the key characteristics of ideal molecular representations and provide a detailed analysis of the existing representations employed in molecular optimization.

#### 4.1.1. Informative Molecular Representations

An ideal molecular representation should be highly informative, which will enable optimization methods to capture abundant molecular features. Molecular sequence representations, such as SMILES and SELFIES, have been extensively utilized in drug design due to their simplicity and interpretability. Cheng et al. [62] introduced Group SELFIES, a fragment-based molecular representation method designed to effectively capture chemical motifs and structural flexibility through string encoding. However, these representations often lack detailed structural information, leading to significant structural variations, even with minor sequence changes [63]. In contrast, graph-based representations offer greater robustness by efficiently encoding chemical interatomic connectivity. Despite this advantage, molecular graphs fail to capture certain critical features, such as the bond angles between atoms. Molecular image representations provide richer information by incorporating bond angles and positional information [64], yet they still fall short of fully representing molecules, which are inherently 3D quantum mechanical objects [65]. While 3D representations include spatial information that better reflects molecular geometry, the added complexity of 3D data significantly increases the computational challenges associated with model learning [66].

#### 4.1.2. Modifiable Molecular Representations

Ideal molecular representations should facilitate easy modifications while preserving the chemical validity of molecules. The molecular SMILES representation is simple and easy to learn; however, it does not guarantee molecular validity, as minor changes in a single character can lead to significant structural modifications [34]. Although some researchers have attempted to address this issue by incorporating grammatical constraints into the encoding–decoding process, they still struggle to ensure the validity of the generated molecules [67]. In contrast, SELFIES [28] achieves 100% validity by separating information related to branches and rings, but it falls short when representing complex, crystalline, and large molecules [68]. Graph-based molecular optimization methods can generate molecules with high validity; however, their modeling processes are more complex than those of sequence-based methods [69]. Image-based molecular optimization encounters challenges in generating valid structures from optimized images. Additionally, 3D molecular representations face difficulties in capturing translation, rotation, and reflection invariance.

### 4.2. Appropriate Datasets

The availability of appropriate datasets poses a significant challenge in the development of effective molecular optimization methods. In this subsection, we first categorize the types of molecular data used across different optimization methods. Then, we review widely adopted molecular datasets and discuss key considerations associated with data acquisition and utilization.

#### 4.2.1. Types of Molecular Datasets

Existing molecular optimization methods typically rely on two types of datasets: paired and unpaired molecular datasets. Unpaired molecular optimization methods often require two sets of molecules with low and high properties or a single set of molecules exhibiting low properties [37,39]. In comparison, paired molecular optimization methods necessitate datasets consisting of numerous molecular pairs, where each pair includes two similar molecules with distinct property values, one with a low property and the other with a high property.

The molecular datasets employed in molecular optimization methods are often sourced from public databases [70,71], for example, the ZINC database [72], which provides 3D molecular structures for virtual screening applications; the ChEMBL database [73], specializing in bioactive molecules with drug-like properties; the QM9 dataset [74], encompassing small organic molecules with quantum chemical properties; and the GDB-13 database [75], which is the largest publicly available repository of small organic molecules. For unpaired molecular datasets, the molecules in these databases can be filtered based on property values, such as low or high QED values. For paired molecular datasets, both the property values of the molecules and their structural similarity must be considered. For example, Jin et al. [22] constructed a paired QED dataset selected from ZINC, in which one molecule in each pair had a QED value between 0.7 and 0.8, while the other had a QED value between 0.9 and 1, with a similarity score exceeding 0.4. Table 2 provides statistics and descriptions of the databases commonly used in molecular optimization tasks.

#### 4.2.2. Challenges and Suggestions in Obtaining Datasets

**Data quality.** High-quality molecular datasets are crucial for enhancing the performance of molecular optimization methods. However, the quality of molecules is affected by potential errors [76]. To address these issues, data reduction and cleaning techniques are useful for providing reusable and trustworthy data, and they have been employed in drug design to improve data quality [71,77]. For example, Papadatos et al. [78] discussed several molecular data management strategies applied to the ChEMBL database, including enhancing data integrity, flagging outliers, and adding annotations.

**Data** quantity. The scarcity of molecular data impedes the performance of drug design methods, particularly for novel or poorly studied diseases [79,80]. In the pharmaceutical industry, data related to drug and lead candidates are often confidential due to intellectual property protections. To address the challenges posed by limited data, several techniques can be employed. First, data augmentation can efficiently expand the training dataset [81]. Second, meta-learning frameworks facilitate knowledge transfer from tasks with abundant information to those with limited data [82]. Furthermore, privacy-preserving computational methods, such as secure multi-party computing [83], federated learning [84], and differential privacy [85], can be employed to jointly train a model from multiple parties without disclosing the original molecular data [86].

**Imbalanced data.** Molecular optimization methods also encounter the challenge of imbalanced data. For a given protein target, the majority of molecules may exhibit inactivity, with only a small fraction demonstrating activity. Several strategies have been proposed to mitigate this issue. First, resampling and oversampling techniques can be employed at the data level to adjust the proportion of active and inactive molecules [87]. Second, deep learning-based molecular optimization methods can integrate the imbalanced training loss to enhance learning efficacy from datasets with imbalances [88].

### 4.3. Optimization Properties

#### 4.3.1. Common Molecular Properties

**Non-biological activity properties.** Non-biological activity properties are often derived from molecular structures, which can be directly assessed using publicly available tools such as MOSES [71], RDKit [89], TDC [90], and ADMET [91]. Several important non-biological activity properties in molecular optimization are described in Table 3, including QED, logP, PlogP, SA, and similarity. These molecular properties can be calculated by RDkit.

**Biological activity properties.** In practical drug development, biological activity properties are crucial for assessing the activity, inhibition, and binding affinity of molecules to disease targets. Biological activity properties are typically estimated by trained prediction models. Table 3 presents several commonly used activities, i.e., DRD2 activity, GSK3β inhibition, and JNK3 inhibition, which have been integrated on the Therapeutics Data Commons (TDC) platform [90].

Moreover, the interaction between molecules and proteins is crucial for practical protein–ligand design, which is typically evaluated by docking scores obtained from molecular simulation docking platforms [98]. For example, Nigam et al. [97] established three benchmark tasks to optimize the docking scores of molecules with target proteins, including the 1SYH, 6Y2F, and 4LDE proteins (Table 3). It is worth noting that practical drug development often involves numerous other disease-related targets that require consideration. When assessing the biological activity of molecules against novel targets, the property values can be obtained through laboratory experiments. Additionally, the biological activity properties can be predicted by surrogate models trained on molecular data with known values of biological properties. To incorporate the docking scores between molecules and new targets, these scores can be simulated using docking platforms such as AutoDock [98], based on the structures of the protein and the molecule.

#### 4.3.2. Multi-Property Optimization

**Figure 3:**
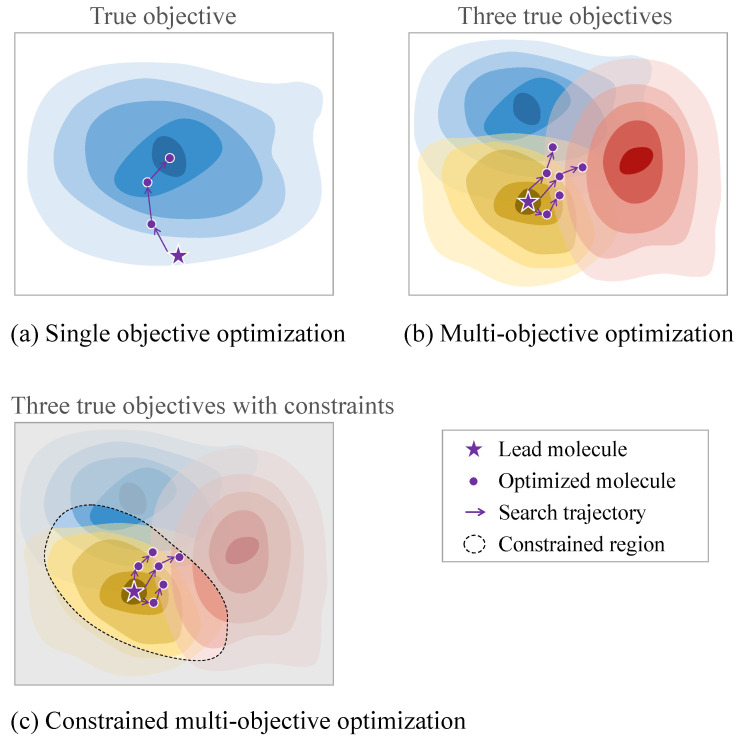
Comparison of the search processes of single-objective, multi-objective, and constrained multi-objective molecular optimization. The darker the colored region, the higher the property. (**a**) Single-objective optimization searches for regions with a high objective value. (**b**) Multi-objective optimization searches for molecules with different trade-offs between three objectives (taking three objectives as an example). (**c**) In constrained multi-objective optimization, the search space is limited by constraints (in gray), and it aims to search for molecules with multiple desired objectives in the constrained region.

As for the aforementioned properties, practical molecular optimization must simultaneously balance multiple conflicting properties. For example, a drug candidate must exhibit desirable drug-likeness, demonstrate effective interactions with disease targets, and possess synthetic feasibility. Furthermore, practical molecular optimization often incorporates further constraints, such as adherence to specific molecular descriptor thresholds or compliance with predefined structural rules [99]. Consequently, molecular optimization is an inherently constrained multi-objective optimization problem that encompasses various objectives and constraints. For multi-objective optimization problems, since multiple properties are in conflict with each other, there is no single molecule with the highest value of all properties but rather a set of Pareto molecules with different preferences for various properties [100]. Moreover, the introduction of additional constraints renders certain regions of the chemical space infeasible, thereby increasing the complexity of the exploration process. Figure 3 visually contrasts the search processes for single-objective, multi-objective, and constrained multi-objective optimization.

To effectively address multi-property optimization challenges, Pareto-based optimization has emerged as a robust framework. This approach avoids the need for assumptions regarding the relative importance of properties and generates a diverse set of Pareto-optimal molecules [101]. Such molecules have been successfully employed in various methods for screening and identifying desired candidates [60].

#### 4.3.3. Challenges in Practical Molecular Optimization Tasks

In real-world drug design, candidate molecules must simultaneously satisfy multiple critical property requirements, including the specific binding affinity to target proteins, synthetic feasibility, good solubility, appropriate blood–brain barrier permeability, low toxicity, and minimal side effects. Beyond these quantifiable optimization objectives, molecular structures must also meet stringent drug-like constraints, such as compliance with Lipinski’s Rule of Five, the avoidance of structural alerts, and the maintenance of a reasonable molecular weight. These multiple requirements make molecular optimization an inherently complex constrained multi-objective optimization problem. Notably, these properties often exhibit intricate interrelationships and trade-offs, making it extremely challenging to identify a single molecule that is optimal across all attributes. Instead, the solution typically involves identifying a set of Pareto-optimal solutions that represent the best possible compromises among competing properties.

For the multi-property optimization challenges, Pareto-based optimization methods have demonstrated significant advantages and are considered the most robust multi-property optimization frameworks. These methods do not require predefined weights of relative importance among properties and can generate a diverse set of candidate molecules. Furthermore, in practical applications, the number of properties to be optimized may exceed four, and additional properties may be introduced over time. In these scenarios, the chemical space containing the desired molecules shrinks considerably, which increases the difficulty of optimization. Traditional Pareto optimization methods often struggle to generate and select high-quality molecules under multi-property optimization. To address these challenges, intelligent generation strategies can be designed to dynamically adjust optimization priorities based on the optimization status of each property, and more efficient selection strategies can also be developed to identify high-quality candidates. For dynamic molecular optimization problems, progressive optimization frameworks offer flexible solutions to accommodate emerging optimization requirements. For instance, DyMol [102] initiates optimization with a single objective and incorporates additional objectives over time, which decomposes complex multi-objective problems into manageable sub-problems for incremental optimization.

In drug design practice, the synthetic feasibility of generated molecules serves as a critical determinant of whether an optimization model can be practically applied in real-world development. Current AI-driven molecular optimization methods predominantly employ synthetic accessibility (SA) [94] scores to evaluate the synthesis of molecules. However, a significant gap persists between these computational scores and the actual synthetic feasibility. To generate molecules that can be reliably synthesized in laboratory settings, on the one hand, it may be useful to train sophisticated deep generative models on extensive databases of known synthesizable molecules to learn synthetic rules; on the other hand, domain-specific knowledge and expert experience from medicinal chemistry should be incorporated during model training to compensate for the limitations of purely data-driven approaches.

In the field of computer-aided drug discovery, structure-based molecular design plays a pivotal role in the development of therapeutic agents for specific diseases. This approach enables the efficient design of candidate molecules with a high binding affinity to target proteins by exploring vast chemical spaces. Notably, leveraging protein–ligand interaction information can significantly enhance the pharmacological activity of generated molecules. For example, PMDM [103] incorporates protein structural information as generation constraints, which establishes a conditional equivariant diffusion model that simultaneously considers both local atomic interactions and global molecular dynamics. The practical utility of PMDM was demonstrated through case studies on two critical drug targets. In a CDK2 lead optimization study, the researchers synthesized molecules generated by PMDM and validated their significantly improved inhibitory activity against CDK2 through in vitro assays. These results demonstrate the practical value of structure-based molecular generation technology in real-world drug development scenarios.

### 4.4. Optimization Algorithms

To enhance molecular optimization, various emerging techniques can be employed to design novel and effective optimization methods. In this subsection, we introduce several promising techniques that provide valuable guidelines for future molecular optimization.

First, practical molecular optimization can be formulated as a constrained multi-objective optimization problem, which aims to find molecules with high properties while satisfying constraints. While most existing methods overlook constraints, making it challenging to generate drug-like molecules, constrained multi-objective optimization algorithms can systematically explore feasible chemical spaces to produce constrained Pareto fronts [104].

Second, molecular optimization methods can be significantly enhanced by leveraging the complementary strengths of different molecular representations. Multi-modal learning, which integrates diverse representations, enables the capture of richer implicit information, thereby improving the robustness and accuracy of optimization [105]. For instance, Luo et al. developed a method that effectively combines 2D and 3D molecular data to extract more comprehensive chemical knowledge [106]. Moreover, within real-world drug discovery pipelines, the incorporation of target protein structural or functional data is critical to computationally guide the generation of bioactive molecules exhibiting specific binding interactions [107].

Third, advanced AI techniques hold potential to enhance molecular optimization [108]. For example, active learning can reduce the labeling cost by iteratively selecting the most informative samples for model training, and it has been used to predict biological activity and target–ligand interactions [109]. Furthermore, transfer learning enables the transfer of knowledge from well-studied tasks to related but data-scarce tasks, making it particularly suitable for molecular optimization in novel diseases with limited datasets [110]. Moreover, multi-task learning can mitigate bias and overfitting by simultaneously training on different tasks in a single model [111], and it can be integrated with existing molecular optimization methods. In addition, with the advancement of large language models (LLMs), integrating LLMs with molecular optimization enables the enhanced extraction of molecular information, the robust capture of chemical rules, and effective knowledge transfer, thereby facilitating the multi-property optimization of molecules [103,112].

## 5. Conclusions

This review provides a comprehensive analysis of existing research in molecular optimization. We first explicitly formulate definitions of molecular optimization problems and highlight their significance in drug discovery and development. We then comprehensively categorize and analyze existing AI-aided optimization models based on the chemical spaces that they explore and the optimization methods that they employ. Furthermore, we discuss the challenges and future prospects associated with the application of molecular optimization models. Thus, this review offers potentially beneficial recommendations for the advancement of AI-based molecular optimization approaches.

## Figures and Tables

**Table 2:** Common datasets and statistics for molecular optimization tasks.

Dataset	Description	Amount	Website
ZINC	Free database of commercially available compounds for virtual screening	>750,000,000	https://zinc15.docking.org/ (accessed on 9 April 2025)
ChEMBL	A manually curated database of bioactive molecules with drug-like properties	2,300,000	https://www.ebi.ac.uk/chembl/ (accessed on 9 April 2025)
PubChem	Largest collection of freely accessible chemical information	119,000,000	https://pubchem.ncbi.nlm.nih.gov/ (accessed on 9 April 2025)
MOSES	Benchmark platform for training process of standardized molecular generation model	1,940,000	https://github.com/molecularsets/moses (accessed on 9 April 2025)
QM9	Molecules with up to 9 heavy atoms	133,885	http://quantum-machine.org/datasets/ (accessed on 9 April 2025)
GDB-13	Small organic molecules database	977,468,314	https://gdb.unibe.ch/downloads/ (accessed on 9 April 2025)
GDB-17	Small organic molecules database	50,000,000	https://gdb.unibe.ch/downloads/ (accessed on 9 April 2025)
QED Pairs	Similar molecule pairs with low and high QED values	88,000	https://github.com/wengong-jin/iclr19-graph2graph (accessed on 9 April 2025)
PlogP Pairs	Similar molecule pairs with low and high PlogP values	99,000	
Drd2 Pairs	Similar molecule pairs with low and high Drd2 values	34,000	

**Table 3:** Common non-biological activity molecular properties for optimization.

Properties	Descriptions
Quantitative estimate of drug-likeness (QED) [92]	A comprehensive index that quantifies the drug-likeness of a molecule as a value between 0 and 1, calculated by combining eight physical descriptors.
Octanol–water partition coefficients (LogP) [93]	A metric assessing the dissolution and diffusion of molecules in the human body through their combined water and lipid solubility, reflecting the membrane absorption capacity.
Penalized logP (PlogP) [58]	The logarithm of the partition ratio of the solute between octanol and water minus the synthetic accessibility score and the number of long cycles.
Synthetic accessibility (SA) [94]	Quantification of the difficulty of synthesizing small molecules in the laboratory on a scale ranging from 1 to 10, where a lower score indicates easier synthesis.
Similarity [23]	Similarity between the lead molecule and the optimized molecule. Tanimoto similarity is widely employed in existing molecular optimization studies due to its computational efficiency.
DRD2 activity [95]	The predicted biological activity score against the dopamine receptor D2 target.
GSK3β inhibition [96]	The estimated inhibition score against the glycogen synthase kinase-3 target.
JNK3 inhibition [96]	The estimated inhibition score against the c-Jun N-terminal kinase-3 target.
1SYH [97]	The docking score of a molecule and an ionotropic glutamate receptor that is associated with neurological and psychiatric diseases.
6Y2F [97]	The docking score of a molecule and the main protease of SARS-CoV-2 that is responsible for the translation of the viral RNA of the SARS-CoV-2 virus.
4LDE [97]	The β2-adrenoceptor GPCR receptor that spans the cell membrane and binds adrenaline, a hormone that mediates muscle relaxation and bronchodilation.

## Data Availability

No primary research results, software, or code was included, and no new data were generated or analyzed as part of this review.
